# A Profession for the Blind

**Published:** 1922-02

**Authors:** F. Chaplin Hall

**Affiliations:** Secretary


*To the Editor of* THE HOSPITAL AND HEALTH REVIEW.

### A Profession for the Blind.

*To the Editor of* THE HOSPITAL AND HEALTH REVIEW.

SIR,—Will you be so good as to allow me space in your valuable columns to state that it was through the untiring efforts of the late Sir Arthur Pearson, Bart., G.B.E., whose tragic death has aroused world-wide sympathy, that blinded soldiers and civilians were trained in the profession of massage?

Might we suggest that there is no way in which the medical profession can better pay lasting tribute to the memory of the late Sir Arthur Pearson (who was President of the Association of Certificated Blind Masseurs) than by employing those who have received training in the massage profession as a result of his indefatigable efforts, and for whom one of his most earnest wishes was that they should be helped to become independent?

With this idea in mind, we should like to draw attention to the existence of our members, who are established in all parts of London and the provinces, and whose names and addresses can be obtained on application to me at 224-6-8, Great Portland Street, London, W. 1 (telephone: Langham 2542), and—since our members do not undertake the treatment of patients without the consent and advice of a registered medical practitioner—to appeal most earnestly for the active support of the medical profession on their behalf.

Yours faithfully,
**The Association of Certificated Blind Masseurs,**
F. Chaplin Hall,
*Secretary.*

